# It Takes a Village: Health and Old-Age Support in China

**DOI:** 10.21203/rs.3.rs-4646034/v1

**Published:** 2024-07-22

**Authors:** Jaqueline Oliveira, Amanda Kerr

**Affiliations:** Rhodes College, Memphis TN, USA.; University of Maryland, College Park MD, USA

**Keywords:** Inter-generational transfers, Private transfers, Old-age support, Health, D64, J14

## Abstract

How do older individuals cope with health impairments and the potential economic losses that ensue? Exploiting longitudinal data on a nationally representative sample of Chinese seniors, we investigate whether and how familial economic support responds to sudden and sizable changes in health. We find that both financial and instrumental support from children go up following a health shock. Furthermore, financial transfers coming from siblings, other relatives and friends increase by a larger percentage than those from children. We also find evidence that instrumental care received from a spouse responds strongly to health shocks. Finally, we find that although labor supply and earned income drop considerably, there is no significant change in non-health expenditure. Our results suggest that households are able to cope with some of the adverse economic impacts of health shocks by relying on support networks that extend beyond their children and grand-children. This is particularly relevant in the context of shrinking family sizes resulting from stringent family planning policies.

## 1 Introduction

The world’s population is ageing at a fast pace, raising many concerns about how to meet the material needs and the healthcare demands of the oldest in society. This challenge is particularly visible in the case of China, a country that houses the world’s largest population over the age of 60, and expects the old-age dependency ratio to increase to a whopping 55% in the next thirty years (Chen et al., 2022).^1^ Despite improvements over the last few decades, nearly 40 million older Chinese had disabilities that affected their activities of daily living as of 2019 (Ai et al., 2022).^2^

Against this backdrop, we ask: how do older Chinese individuals cope with economic losses in the face of severe health impairments? More specifically, we investigate the role of intergenerational and inter-household transfers. The focus on family support is warranted in the Chinese context: despite there having been major healthcare reforms since 2009, which significantly expanded coverage, the pension system available to rural registered hukou holders provides roughly one-fourth of the poverty line.^3^ It is not surprising, therefore, that family members are still viewed as the main source of care in old age (Sun, 2002; Wu and Li, 2014; Ai et al., 2022; Chen et al., 2022). However, the shrinking family sizes resulting from China’s stringent family planning policies and the erosion of filial piety values cast doubt on whether support from adult children alone can meet old-age demands.

To answer the question posed above, we rely on data from four waves of the China Health and Retirement Longitudinal Study (CHARLS), which is a nationally representative sample of Chinese individuals aged 45 and older, and their spouse, spanning the years 2011, 2013, 2015, and 2018. We measure sudden and large changes in health by using an individual-level health index created from self-reported measures of difficulties with six types of activity of daily living (ADL): getting dressed, bathing or showering, eating, getting in and out of bed, using the toilet and controlling urination and defecation.^4^ The main identifying assumption we make is that the *within-individual* short-term (two- to three-year) variation in the ADL index is uncorrelated with short-term changes in the unobservable determinants of transfers. We argue that our identification assumption is plausible particularly because we control for village/community-year fixed effects so that any time changes in an individual’s environment—such as weather shocks and healthcare policy changes—are held constant. We also establish that the within-individual wave-to-wave variation in the health index is uncorrelated with the death of their children, siblings, and parents, suggesting the absence of common health shocks affecting members of the same family who possibly live in close proximity (that is, same village or community).

Our main finding is that both the likelihood of receiving financial support and the amounts of family-provided financial support increase following a health shock. In particular, a one-unit increase in the ADL index—which is a change from reporting no difficulties with ADLs to reporting some difficulty with all six basic ADLs—leads to a 5.3 ppts increase in the likelihood of receiving financial assistance, and a 42% increase in the amount of transfers received. When we break it down by source, we find that both transfers from children and from siblings (and other relatives and friends) respond to health shocks, but transfers from parents do not. We also find that transfers from siblings and other relatives and friends are *more* responsive to health shocks than those coming from children and grand-children.

Looking beyond financial transfers, we investigate the effect of a health shock on family-provided instrumental care for those who reported difficulties with at least one basic ADL or instrumental ADL.^5^ We see an increase in time transfers from spouse and from children, but not from siblings and other relatives. And while spouses are the main providers of instrumental care, time assistance from children is more responsive to health shocks than time assistance from a spouse. Finally, consistent with results from previous studies, we find that health shocks lead to sizable decreases in labor supply and earned income, although we see no impact on spouse labor supply. Despite the adverse effect on earned income, we find no statistically significant changes in non-healthcare expenses, suggesting that older households are able to insure their consumption against health shocks.

This paper relates to a well-established literature that investigates how health-related shocks impact economic outcomes such as labor and income (Beegle, 2005; Lindelow and Wagstaff, 2005; Yamano and Jayne, 2004; Khan et al., 2015; Liu, 2016; Dobkin et al., 2018; Wang et al., 2023), consumption, health and non-health expenses (Dercon and Krishnan, 2000; Gertler and Gruber, 2002; Wu, 2003; Asfaw and Braun, 2004; Mitra et al., 2016; De Weerdt and Dercon, 2006; Gertler et al., 2009; Islam and Maitra, 2012; Finkelstein et al., 2013; Wagstaff and Lindelow, 2014; Šedivý, 2023; Blundell et al., 2024), and spousal labor supply (Jeon and Pohl, 2017; Macchioni Giaquinto et al., 2022; Jolly and Theodoropoulos, 2023). Other studies find evidence of family transfers as a coping mechanism to health shocks in developing (Genoni, 2012; Sparrow et al., 2014; Dureja and Negi, 2024) and developed country context (Schaller and Eck, 2023). Our paper departs from these previous studies in important ways. First, we are able to zero in on the *source* of transfers, so we can answer questions pertaining the role of adult children versus other family support networks in the provision of old-age financial support. Second, we rely on measures of difficulties with basic ADLs to create our measure of health shock, which is an arguably more objective way to capture sizable changes in health status (Gertler and Gruber, 2002). Finally, we focus on a sample of older individuals and are, therefore, able to shed light on coping mechanisms to address the challenges faced by ageing societies.^6^ We argue that these mechanisms are even more relevant within the context of a decrease in the number of adult-child caretakers and the still widespread expectation among the Chinese that children be the main providers of old-age support.

Lastly, this study also relates to a vast literature that examines the determinants of informal caregiving and financial transfers from adult children to elderly parents in both developing and developed country contexts (Rosenzweig, 1988; McGarry and Schoeni, 1995; Konrad et al., 2002; Checkovich and Stern, 2002; Sloan et al., 2002; Cai et al., 2006; Byrne et al., 2009; Davies, 2011; Antman, 2012; Lei et al., 2015; Horioka et al., 2018; Ho, 2019). Our study differs from those by looking into the relationship between health and old-age support.

The next section presents a simple theoretical framework that we use to motivate our empirical exercise. Section 3 describes the CHARLS data in detail. Section 4 outlines the empirical approach to estimating the causal impact of health on familial support. Section 5 presents the main findings and further evidence on the broad economic consequences of health shocks. Section 6 offers concluding remarks.

## 2 Motivating framework

In this section, we present a simple model to help us understand how a shock to health could impact family-provided financial assistance. Consider two agents, a donor i and a recipient j. Each agent derive utility from consumption goods according to utility functions $u\left(c_{i}\right)$ and $v\left(c_{j}\right)$. Assume that $u^{\prime }(\cdot )>0$.0 and $v^{\prime }(\cdot )>0$, and that $u^{\prime \prime }(\cdot )<0$ and $v^{\prime \prime }(\cdot )<0$. Let $y_{i}$ and $y_{j}$ be donor and recipient income, respectively, and $t_{ij}$ be the transfers made from donor i to recipient j. And assume further that the donor is altruistic towards the recipient, so that they choose the level of transfers that maximize the sum of the two utilities as follows:^7^

$$\begin{array}{ll} \underset{c_{i},c_{j}}{max}\; & u\left(c_{i}\right)+v\left(c_{j}\right)\\ \text{s.t.}\; & c_{i}=y_{i}-t_{ij},\\ & c_{j}=y_{j}+t_{ij},\\ & t_{ij}\geq 0 \end{array}$$

It follows from the first-order conditions that, if donor’s income is sufficiently lower compared to recipient’s, such that $u^{\prime }\left(y_{i}\right)>v^{\prime }\left(y_{j}\right)$, we have a corner solution and $t_{ij}^{*}=0$. If donor’s income is sufficiently larger, such that $u^{\prime }\left(y_{i}\right)<v^{\prime }\left(y_{j}\right)$, we have an interior solution and the utility-maximizing amount of transfer is implicitly determined by the following equation:

$$u^{\prime }\left(y_{i}-t_{ij}^{*}\right)=v^{\prime }\left(y_{j}+t_{ij}^{*}\right)$$

Now we can ask: what happens to transfers from i to j when j experiences a (negative) health shock? On the one hand, the health shock would impact labor productivity, leading to a decrease in recipient’s income, $y_{j}$. This, in turn, would increase $t_{ij}^{*}$ in order to equalize the marginal utilities of donor’s and recipient’s consumption (we call it a productivity shock). On the other hand, the change in health status could affect the recipient’s preferences–if for example individuals start to enjoy consumption of goods or services less when they get sick and/or face mobility constraints—and shift $v^{\prime }(.)$ downwards (we call it a preference shock). If the recipient’s marginal utility of consumption is lower for all levels of $c_{j}$ as a result of the health shock, then we should see a decrease in $t_{ij}$.^8^ Because one cannot know if the productivity shock is larger than the preference shock, it follows that a shock to health has a theoretically ambiguous impact on transfers. Our empirical analysis is able to shed light on the overall effect of a health shock on transfers, as well as the changes in consumption patterns. It is worth pointing out, however, that testing the validity of this simple model against other potential transfer motives is beyond the scope of this paper.

## 3 CHARLS Data

We rely on a nationally representative sample of Chinese individuals older than 45 and their spouse (whenever one is present) from waves 2011, 2013, 2015, and 2018 of the China Health and Retirement Longitudinal Study (CHARLS).^9^ Our empirical analysis uses data from the Harmonized CHARLS dataset developed by the Gateway to Global Aging Data.^10^

After missing data on key variables of interest are dropped, we are left with a total of 30,989 individual-year observations.^11^ The data cover a total of 16,302 individuals who reported having at least one living child and who are at least 40 years of age at the time of the survey. Out of the 16,302 individuals in our estimating sample, 48% are males, 61% live in rural areas, only 11% have some level of education, and 87% report living with a spouse. Furthermore, they are 57.9 years old on average and have an average of 2.7 children.

To measure sizable and sudden changes in health, we follow Gertler and Gruber (2002) and utilize individual-level data on reported difficulties with activities of daily living (henceforth ADLs).^12^ We consider six basic ADLs: getting dressed, bathing and showering, eating, getting in and out of bed, using the toilet, and controlling urination and defecation. For each ADL, the respondent is assigned a value one if they reported some difficulties with the activity, and zero if no difficulties.^13^ We then create an index by adding up all the six ADL measures and dividing the result by six. Therefore, the health index ranges from 0 (if one has no difficulties with any of the six activities) to 1 (if one has difficulties with all of the six basic ADLs).

Table 1 summarizes the health measure. The ADL index is positive for about 20% of the estimating sample, and the (previous year) unconditional average ADL index is 0.06. Conditional on a positive ADL index, the mean ADL index is 0.33, which indicates difficulties with two (out of six) basic ADLs, on average. The year-to-year change in the ADL index is very close to zero, 0.01 on average. This is due in large part to the prevalence of zero values in the ADL index across years. But even when we condition on non-zero changes in the ADL index, the average change in the ADL index is only 0.05. Furthermore, the next two indicator variables measuring the percentages of positive and negative changes in the ADL index among the observations in the estimating sample show that 11% of the non-zero changes that occurred were improvements in the ADL index (that is, ADL index decreased), whereas 14% of the non-zero changes were deterioration in the ADL index. Only 3% of the observations were cases where there were no changes in a bad health state. These facts suggest that the variation in the health measure are not coming predominantly from individuals who experience permanent health deterioration, but also from ones that recover from adverse health states.

Turning now to the main outcome variable of interest, we describe the measures of family-provided financial support available in the CHARLS. The survey asks the family respondent whether they or their spouse received economic assistance in the past year and, if so, how much they received. This economic assistance could be monetary or (the value of) in-kind support (such as food, clothes, etc.). Furthermore, it includes regular transfers (living expenses, water, electricity, telephone, loan payments, mortgage/rent) and non-regular transfers (money for major festivals, birthdays, weddings, and funerals). We carry out our analysis separately by three sources of assistance: from children and/or grand-children, from parents and/or parents in law, and from siblings, other relatives and/or friends.^14^

Table 2 summarizes the financial transfer variables across the individual-year observations that make up our estimating sample.^15^ From the summary statistics, it is evident that family support is prevalent among study subjects. For 81% of the observations, the amount of transfers received from children, parents, and/or other relatives and friends was positive. Most transfers come from children/grand-children: around 77% received transfers from children and/or grand-children, 9% from parents and/or parents-in-law, and 21% from siblings, other relatives, and friends.^16^

The average amount of transfers received annually is around 4.4 thousand yuan (in 2010 value), which is roughly 700.00 U.S. dollars.^17^ When we break it down by source, we see that the average amounts received from children/grand-children are larger than that received from siblings, other relatives and friends (3,469 yuan from children compared to 849 yuan from other relatives). However, when we condition on positive transfers, the average amount received from children/grand-children (4,512 yuan) is comparable to the average amount received from siblings, other relatives and friends (4,028 yuan). This illustrates the relevance of familial support networks other than children.

## 4 Empirical Strategy

To estimate the causal impact of health shocks on transfers (and other outcomes), we follow Gertler and Gruber (2002) and propose the following estimating equation:

(1) $$\Delta Y_{ict}=\alpha_{0}+\alpha_{1}\Delta {Health}_{ict}+\alpha_{2}X_{i}+\alpha_{ct}+\varepsilon_{ict},$$

where i indexes the individual, c the location (urban community or rural village), and t the survey year. ${\text{Health}}_{ict}$ is a health index that measures difficulties with basic ADLs; a value 0 indicates that the individual can perform all basic ADLs, whereas a value 1 indicates that the individual cannot perform any of the basic ADLs.^18^ $X_{i}$ is a vector of demographic controls: sex, year of birth indicators, educational level indicators, and rural/urban status.

The parameter $\alpha_{ct}$ represents village-year (or urban community-year) fixed effects and it captures any location-year specific shocks to individuals’ health status that would also determine their households’ economic outcomes. For example, any year-to-year weather-related event that adversely affects the local economy and simultaneously causes health problems in the population would be captured by the village-year fixed effects. Furthermore, any changes in health-care policies over time that happen at the local level—and that could be correlated with local economic conditions that also determine the individual’s needs for economic support-are controlled for.

Finally, and more importantly, because we employ a first-differences approach, our estimates control for unobservable time-invariant individual heterogeneity. The reliance on within-person year-to-year variation in ADL limitations allows us to control for time-invariant unobservable attributes such as health endowments, habits, and tastes that would be systematically correlated with socio-economic outcomes and living standards. One could argue for example that, in the cross-section, parents in worse health receive less transfers from family members because these family members are also more likely to have shared their poor health and are, therefore, less able to provide economic support to others. This source of bias would be taken care of by estimating the model in first differences.

One possible source of bias would arise if short-term, year-to-year, variations in the health of people in the same family are systematically correlated when these family members live in close proximity and are exposed to common village or community-level shocks that impact their health and economic status. Because our models include interactions between village/community and year fixed effects, such threats to identification from family members living in close proximity are less concerning. Furthermore, we later provide evidence that our measure of health shock is uncorrelated with family mortality. (See Table 6.)

If one conceptualizes inter-household transfers as a form of informal insurance, two issues arise in interpreting the estimates from the model in the equation above. One is the adverse selection problem: past investments in stronger family ties and fostering of inter-household networks may have been motivated by poorer health, which may lead to unhealthier individuals being more likely to receive transfers. We argue, however, that to the extent that these investments happen over the long run, controlling for individual fixed effects and exploiting year-to-year variations in ADL limitations mitigate biases coming from this source.

The other issue is the moral-hazard problem: individuals who are part of informal insurance networks may alter their health behaviors and habits over time in response to the expectation of future transfer receipt. This could lead to a reverse causality bias, whereby the increase in transfers leads to health deterioration. We offer two answers here. First, we are using an ADL-limitation measure that captures the onset of severe health limitations with basic tasks like bathing, eating, and dressing. Therefore, it would be unlikely that short-term changes in behaviors and habits would provoke the kind of sudden and drastic health changes being measured by our health index variable. Second, even if it did, we would expect that the source of the transfer would not matter for these changes in behavior triggered by a moral-hazard problem. However, it is not what we find in the data (since there were no changes in transfers coming from parents or in-laws).

Another concern would be that other unobservable time-varying idiosyncratic events could be driving both transfers and health. One example would be if an individual became unemployed. This could simultaneously increase transfers (by lowering income) and reduce investments in health and/or lead to negative health behaviours. We argue, however, that these changes in health investment and/or behaviour would have to be too drastic to plausibly lead to an individual being unable to perform basic ADLs in a short time span.

In summary, it is unlikely that these sources of bias—correlated family health, adverse selection, and moral hazard—explain the findings generated from the model in Equation (1) when the estimates rely on short-term, within-individual variation in a health index created from limitations in basic ADL, and the model controls for common shocks to health and material well-being at the local level by including community/village-year fixed effects.

## 5 Findings

### 5.1 Family-provided financial support

The findings in this section answer the following question: How does family support change in response to short-term variations in an individual’s ability to carry out basic daily-living tasks? We begin by focusing on measures of family-provided financial support. Table 3 presents results for financial assistance from non-resident children/grand-children and parents/in-laws, and Table 4 shows results for financial support from other non-resident relatives such as siblings, other relatives and friends. We show results for the entire sample (All) as well as by status (Rural vs Urban) and respondents’ sex (Female vs Male). The column labelled “Mean” shows the mean of the dependent variable in the previous survey year. When interpreting all results, we will call “health shock” a change in the health index from 0 to 1, that is, an individual going from being able to perform all six basic ADLs to having some difficulty with all six basic ADLs. Additionally, when interpreting results from log-level specifications, we apply the following transformation to obtain the exact percent change in the outcome variable: $\left[exp\left({\overset{ˆ}{\alpha }}_{1}\right)-1\right]\;*\;100$. Lastly, standard errors are clustered at the household level.

Our estimates show that a health shock leads to a 4.1 ppts increase in the likelihood an individual receives financial assistance from children and/or grand-children. Because around 61.1% of respondents received transfers from children in the previous period, this effect is equivalent to a 6.7% increase in the likelihood of receiving financial assistance from children. Our results also point to a 34.5% increase in the amount of transfers received from children; when we consider the amount of transfers divided by the number of children, we estimate a 26.7% increase in transfers receipt. The estimates presented by sub-sample reveal differences between rural and urban settings: while there is significant increases in transfers received from children among rural dwellers, the estimated effects for the urban sample are smaller and not statistically significant. Finally, transfers received from parents and/or parents in law do not respond to changes in health. (It is worth noting that transfers from parents are relatively uncommon, which is not surprising considering that our sample is comprised of older individuals.)

Turning to the results from Table 4, we find that financial support from siblings, other relatives and/or friends responds strongly to health shocks. In particular, we estimate a 6.5 ppts increase in the likelihood of receiving transfers from siblings, other relatives and friends. And considering that only 14.1% of individuals received transfers from this family group in the previous survey year, the effect corresponds to a 47% increase in transfer receipt. They also experience a 49% increase in the amount of financial support coming from siblings, other relatives, and friends. The results by sub-sample show similar patterns by rural/urban status and by sex. Finally, when we consider all family network combined, we estimate a 42% increase in the total amount of financial transfers from all sources.

### 5.2 Other forms of family support

Table 5 presents estimates for non-financial and instrumental support from family. The first outcome is household size, which refers to the number of household members other than the spouse (when one is present). We do see a slight increase in average household size associated with a health shock, but the change is coming mostly from the urban sub-sample.

The second outcome is an indicator for the presence of at least one co-resident child. We understand that co-residence might not necessarily measure help that children provide to ageing parents, but it could represent the opposite. However, we argue that changes in the likelihood of co-residing with a child associated with *changes in ADL limitation* are more likely to capture support from children to parents in need of health care. The results show no statistically significant changes in co-residence with children. This suggests that the effects we find regarding transfers from children are not understating a change in the *amount* received because of lower numbers of non-resident children.

The next outcome variables are an indicator for living in the same city or county as (at least one of) their children, an indicator for any weekly contact (in person or virtual) with children, and any weekly in person contact with parents. While the majority of the sample live near children (89%) and have contact with them (92%), as shown by the averages of the dependent variables, there is virtually no change in those outcomes following a health shock. In particular, the variable that captures geographic proximity to children suggests that they are not moving to the same city or county as their parents to deal with changes in parental health. ^19^

Finally, the table presents results for various measures of family-provided care with ADLs and IADLs. These variables are non-missing only for individuals who reported difficulties with at least one ADL or one IADL.^20^ We see that around 70.3% of the individuals who needed help with an adl/iadl received help. And the estimates show a 8.4 ppt increase in the likelihood of getting help with an adl/iadl when the health index increases by one unit.

When we break it down by source of help, we find that spouses are the main providers of adl/iadl care: 60.9% received help from spouse compared to 31.1% from children and 4.2% from other relatives. ^21^ Nonetheless, the likelihood an individual receives adl/iadl care from children increases by 12.7 ppts in the whole sample, whereas the likelihood of receiving care from a spouse goes up by less, 7.9 ppts. Furthermore, there is a large and significant increase of 6.2 in the number of days per month an individual receives help with adl/iadl from children.

One relevant concern is the potential changes in the *size* of the family support network that would take place if changes to an individual’s health are correlated with the health of their closest family members. To check for this possibility, Table 6 shows the relationship between an individual’s health index and the number of dead sons and daughters, brothers and sisters, indicators for dead father and mother, and dead spouse. The results show no association between an individual’s health shock and the indicators of family mortality. These findings give us confidence that the short-term, wave-to-wave variation in the health index do indeed capture idiosyncratic changes in health status and the results we find are unlikely to be explained by within-family correlation in idiosyncratic determinants of health.

Another concern is that the health-transfer relationship is reverse: individuals allow for health decays in face of the expectation of future transfers, leading to a negative relationship. Or that higher transfers allow individuals to spend more on health care, leading to a positive relationship. While issues related to reverse causality cannot be completely ruled out, it would be difficulty for these explanations to fully and solely account for the patterns we see regarding the *source* of the transfers: transfers from other relatives or friends bear a stronger relationship with health, and mostly among rural dwellers, not urban ones. Furthermore, if the “expectation of future transfers” explanation relies on individuals being able to learn about the transfer behaviour of others, it is much harder to do so when the transfers are coming from non-resident distant relatives and from friends as opposed to their own children and parents.

### 5.3 Income and consumption

In a framework where donors are altruistic towards transfer recipients, a deterioration in recipients’ health would lead to higher transfers by hindering their ability to work and make income. In Table 7 we look at how a health shock impacts labor supply and earned income.

The dependent variable “Currently working” is an indicator for whether the individual engaged in any work in the past year; “Hours worked” is the number of hours worked per week; “Lost work bc sick” indicates if, conditional on being employed, an individual lost work due to sickness; and “Earned income” is the amount of income from labor sources, in logs. Our results how that all these outcomes are effected by a health shock: a change in the health index from 0 (no difficulties with any basic ADL) to 1 (some difficulty with all six basic ADLs) leads to a 11.3 ppt decrease in the likelihood an individual is working, a 4 hr/week decrease in hours worked, a 34.2 ppt increase in the likelihood of losing work due to sickness, and a 34% decrease in earned income. There are differences by sex and rural/urban status: the adverse effects on labor are more pronounced for those living in rural areas and among male respondents. Interestingly, there is no significant decrease in earned income among female respondents, perhaps reflecting unpaid work.

We also look at whether a shock to one’s health impacts the labor outcomes of their spouse and we find no statistically significant effect on those outcomes. One notable exception is observed among females. For this sub-sample, we estimate a 5 ppts decrease in the likelihood a spouse is currently working. Finally, the last outcome variable in Table 7 is an indicator for whether income Table 6: Health and family mortality from other household members (excluding spouse) is positive. The estimate for the entire sample shows a small but significant 4 ppts increase in the likelihood that other members bring in positive income. This effect is larger among the female sample.

Table 8 presents results for total household per capita income and pension income. The estimates for the entire sample show no statistically significant impact on total income nor pension income. The analysis by sub-sample shows no clear pattern. (And the point estimates in some cases are large but too noisy to distinguish the estimates from zero.) Lastly, to better understand what happens to household material well-being, in Table 8 we investigate how consumption responds to short-term changes in health. We look at three consumption categories: the household per capita consumption of food items (purchased food and food eaten from own production, meals eaten out, and alcohol/tobacco); the household per capita consumption of non-food items in the past month (utilities, fuels, entertainment, transportation, daily items, etc.); and the household per capita consumption of non-food items in the past year (clothing, durable goods, education, travel, maintenance and repairs and, importantly, medical expenses). We find a large and significant *increase* in non-food items that include medical expenses—mostly among the sub-sample of rural residents whose access to health care coverage is less extensive compared to urban dwellers. However, there is no statistically significant impact on the other consumption categories. This might suggest that individuals are able to smooth their consumption in face of health shocks as in Genoni (2012) and Dureja and Negi (2024).

## 6 Final Remarks

In light of the demographic challenges face by China—a growing old-aged population coupled with low birth rates and increasing rural-urban migration of adult children—this paper sheds light on the informal mechanisms older individuals rely on to cope with health-related income losses. We find that rural dwellers receive more financial transfers from non-resident children and non-resident siblings, other relatives and friends, whereas urban hukou holders receive more financial transfers from siblings, other relatives, and friends, but not from children and parents. Both spouse and children provide more support in the form of instrumental care. Finally, we find that, although short-term changes in ADL limitations have a large impact on labor and income, household per capita consumption of non-health related items does not fall, suggesting that some smoothing in consumption takes place. Our findings suggest that, despite the recent expansion in publicly-provided health insurance, senior Chinese households still rely on a network of informal financial and instrumental support that comprises spouse, children, siblings, and other relatives and friends. Therefore, the answer to the question regarding how older individuals cope with health shocks is: “it takes a village”. Whether this reliance on familial support poses challenges to the younger generations that are spending time and financial resources to meet the health needs of ageing family members is subject for future research.

## Figures and Tables

**Table 1:** Summary statistics: ADL index

	Mean	Std. Dev	Min	Max

ADL > 0	0.20	0.40	0.00	1.00
ADL index	0.06	0.15	0.00	1.00
ADL index | > 0	0.33	0.22	0.17	1.00
Change in ADL	0.01	0.17	−1.00	1.00
Change in ADL | ≠ 0	0.05	0.34	−1.00	1.00
ADL index better	0.11	0.31	0.00	1.00
ADL index worse	0.14	0.35	0.00	1.00
No change in bad ADL	0.03	0.18	0.00	1.00
No change in good ADL	0.72	0.45	0.00	1.00

N	30989			

*Notes:* ADL index is created by adding six indicator variables for the presence of difficulties with a basic ADL (getting dressed, bathing or showering, eating, getting in and out of bed, using the toilet, and controlling urination and defecation) and dividing the result by six. Therefore, Health ranges from 0 (if one no difficulties with any of the six basic ADLs) to 1 (if one has difficulty with all six basic ADLs). ADL > 0 is an indicator for postive ADL index. ADL index is the value from the previous survey year. ADL index| > 0 is the value from the previous survey year if the value is greater than zero; otherwise it is set to missing. Change in ADL is the difference between the ADL index in the current year and the previous year. Change in ADL| ≠ 0 is the non-zero difference between current year and previous year ADL index. ADL index better is an indicator that takes on value one if the difference between current and previous-year ADL index is negative. ADL index worse is an indicator that takes on value one if the difference between current and previous-year ADL index is positive. No change in bad ADL is an indicator that takes on value one if the difference between current and previous-year ADL is zero and previous-year ADL is positive. No change in good ADL is an indicator that takes on value one if the difference between current and previous-year ADL is zero and previous-year ADL is zero. Data source: CHARLS 2018, 2015, 2013, 2011.

**Table 2:** Summary statistics: Family-provided financial support

	Mean	Std. Dev.	Min	Max

Got transfers from any source	0.81	0.39	0.00	1.00
Got transfers from children/grandch	0.77	0.42	0.00	1.00
Got transfers from parents/in-laws	0.09	0.28	0.00	1.00
Got transfers from relatives/friends	0.21	0.41	0.00	1.00
Total amt. transfers received all sources	4401.39	8080.02	0.00	96388.16
Amt. transfers from children/grandch	3469.33	6595.58	0.00	96388.16
Amt. transfers from children/grandch| > 0	4512.16	7202.26	0.82	96388.16
Amt. transfers from parents/in-laws	214.50	2046.07	0.00	82236.84
Amt. transfers from parents/in-laws| > 0	2462.02	6523.35	3.29	82236.84
Amt. transfers from relatives/friends	849.88	4209.26	0.00	91726.62
Amt. transfers from relatives/friends| > 0	4028.89	8437.58	4.11	91726.62

N	30989			

*Notes:* Got transfers from any source is an indicator for whether the individual and spouse received any economic support from children/grand-children, parents/in-laws, other relatives and friends in the past year. Got transfers from children/grandch is an indicator for whether the individual and spouse received any economic support from children/grand-children. Got transfers from parents/in-laws is an indicator for whether the individual and spouse received any economic support from parents/in-laws. Got transfers from relatives/friends is an indicator for whether the individual and spouse received any economic support from siblings, other relatives, and friends. Total amt. transfers received is the sum of transfers received from children/grand-children, parents/in-laws, other relatives and friends in the past year. Amt. transfers from children/grandch is the total amount of transfers received from children/grand-children in the past year. Amt. transfers from children/grandch| > 0 is the total amount of positive transfers received from children/grand-children. Amt. transfers from parents/in-laws is the total amount of transfers received from parents/in-laws in the past year. Amt. transfers from parents/in-laws| > 0 is the total amount of ositive transfers received from parents/in-laws in the past year. Amt. transfers from relatives/friends is the total amount of transfers received from siblings, other relatives, and friends in the past year. Amt. transfers from relatives/friends| > 0 is the total amount of positive transfers received from siblings, other relatives, and friends in the past year. Data source: CHARLS 2018, 2015, 2013, 2011.

**Table 3:** The effects of health on financial support from children/grand-children and parents/in-laws

Dep. var.: Change in	All	Mean	Rural	Urban	Female	Male

Got transfers from children/grandch	0.040 (0.019)**	0.611	0.047 (0.022)**	0.018 (0.036)	0.043 (0.024)*	0.051 (0.030)*
Amt. transfers from children/grandch	0.297 (0.141)**	4.592	0.337 (0.160)**	0.165 (0.285)	0.321 (0.180)*	0.377 (0.224)*
Amt. transfers per child	0.237 (0.117)**	3.652	0.270 (0.132)**	0.128 (0.240)	0.268 (0.151)*	0.293 (0.184)
Got transfers from parents/in-laws	−0.013 (0.026)	0.061	0.011 (0.031)	−0.062 (0.050)	0.000 (0.041)	−0.016 (0.046)
Amt. transfers from parents/in-laws	−0.015 (0.163)	0.394	0.135 (0.188)	−0.325 (0.316)	0.072 (0.239)	−0.046 (0.295)

*Notes:* Each observation is an individual-year pair, and the units of analysis are individuals older than 45 and their partners, when one is present, regardless of age. Table shows the coefficients from a regression of the first difference of the dependent variable—displayed in the first column—on the first difference of the health shock measure. **All** shows results for all sample; **Mean** displays the mean of the lagged value of the dependent variable; **Rural** and **Urban** shows results for subsamples of rural and urban households, respectively; **Female** and **Male** shows results for subsamples of female and male respondents, respectively. Health is created by adding six indicator variables for the presence of difficulties with a basic ADL (getting dressed, bathing or showering, eating, getting in and out of bed, using the toilet, and controlling urination and defecation) and dividing the result by six. Therefore, Health ranges from 0 (if one no difficulties with any of the six basic ADLs) to 1 (if one has difficulty with all six basic ADLs). Dependent variables are displayed in the first column. Got transfers from children/grandch is an indicator for whether the individual and spouse received any economic support from children/grand-children. Amt. transfers from children/grandch is the total (log) transfers received from children/grand-children in the past year. Amt. transfers per child is the total (log) transfers received from children/grand-children in the past year divided by the total number of children (living and deceased). Got transfers from parents/in-laws is an indicator for whether the individual and spouse received any economic support from parents/in-laws. Amt. transfers from parents/in-laws is the total (log) transfers received from parents/in-laws in the past year. Logged values are replaced with zeros when the amounts are zero. Monetary values are in 2010 Yuan. All amounts are divided by two (before taking logs) when individual is partnered. All regressions include rural village (or urban community) fixed effects interacted with survey year fixed effects. Demographic controls: sex, education, birth-year indicators, number of children ever born, and rural/urban status. Standard errors are clustered at the household level. Data source: CHARLS 2011, 2013, 2015, and 2018.

* p<0.1,

** p<0.05,

*** p<0.01.

**Table 4:** The effects of health on financial support from siblings, other relatives and friends

Dep. var.: Change in	All	Mean	Rural	Urban	Female	Male

Got transfers from relatives/friends	0.065 (0.020)***	0.141	0.067 (0.024)***	0.061 (0.036)*	0.067 (0.027)**	0.062 (0.031)**
Amt. transfers from relatives/friends	0.399 (0.136)***	0.990	0.390 (0.161)**	0.446 (0.253)*	0.409 (0.178)**	0.380 (0.214)*
Got transfers from any source	0.053 (0.018)***	0.651	0.064 (0.020)***	0.021 (0.036)	0.062 (0.023)***	0.054 (0.030)*
Total amt. transfers received all sources	0.420 (0.138)***	4.974	0.482 (0.153)***	0.237 (0.293)	0.475 (0.175)***	0.445 (0.230)*

*Notes:* Each observation is an individual-year pair, and the units of analysis are individuals older than 45 and their partners, when one is present, regardless of age. Table shows the coefficients from a regression of the first difference of the dependent variable—displayed in the first column—on the first difference of the health shock measure. **All** shows results for all sample; **Mean** displays the mean of the lagged value of the dependent variable; **Rural** and **Urban** shows results for subsamples of rural and urban households, respectively; **Female** and **Male** shows results for subsamples of female and male respondents, respectively. Health is created by adding six indicator variables for the presence of difficulties with a basic ADL (getting dressed, bathing or showering, eating, getting in and out of bed, using the toilet, and controlling urination and defecation) and dividing the result by six. Therefore, Health ranges from 0 (if one no difficulties with any of the six basic ADLs) to 1 (if one has difficulty with all six basic ADLs). Dependent variables are displayed in the first column. Got transfers from relatives/friends is an indicator for whether the individual and spouse received any economic support from siblings, other relatives, and friends. Amt. transfers from relatives/friends is the total (log) transfers received from siblings, other relatives, and friends in the past year. Got transfers from any source is an indicator for whether the individual and spouse received any economic support from children/grand-children, parents/in-laws, other relatives and friends in the past year. Total amt. transfers received is the sum of transfers received from children/grand-children, parents/in-laws, other relatives and friends in the past year, in logs. Logged values are replaced with zeros when the amounts are zero. Monetary values are in 2010 Yuan. All amounts are divided by two (before taking logs) when individual is partnered. All regressions include rural village (or urban community) fixed effects interacted with survey year fixed effects. Demographic controls: sex, education, birth-year indicators, number of children ever born, and rural/urban status. Standard errors are clustered at the household level. Data source: CHARLS 2011, 2013, 2015, and 2018.

* p<0.1,

** p<0.05,

*** p<0.01.

**Table 5:** The effects of health on non-financial support from family

Dep. var.: Change in	All	Mean	Rural	Urban	Female	Male

Household size	0.094 (0.056)*	1.578	0.056 (0.070)	0.179 (0.092)*	0.079 (0.073)	0.112 (0.089)
Had children co-residing	0.023 (0.019)	0.576	0.020 (0.024)	0.028 (0.033)	0.023 (0.026)	0.021 (0.030)
Lived near children	−0.011 (0.014)	0.894	−0.014 (0.017)	−0.000 (0.025)	−0.014 (0.019)	−0.007 (0.023)
Had contact with children	0.019 (0.014)	0.922	0.015 (0.018)	0.026 (0.020)	0.025 (0.017)	0.013 (0.022)
Had contact with parents	−0.031 (0.037)	0.562	−0.011 (0.044)	−0.080 (0.069)	−0.035 (0.052)	−0.044 (0.071)
Had ADL help	0.084 (0.030)***	0.703	0.117 (0.036)***	−0.010 (0.056)	0.107 (0.041)***	−0.001 (0.060)
Had ADL help from spouse	0.079 (0.040)**	0.609	0.088 (0.047)*	0.065 (0.076)	0.084 (0.058)	0.018 (0.075)
Days/month spouse helped ADL	1.668 (1.711)	12.777	2.534 (2.078)	−1.581 (3.410)	3.426 (2.335)	−1.188 (3.470)
Had ADL help from children	0.127 (0.034)***	0.311	0.105 (0.039)***	0.199 (0.071)***	0.167 (0.046)***	0.042 (0.065)
N. children helped with ADL	0.653 (0.281)**	0.920	0.493 (0.342)	0.939 (0.481)*	1.014 (0.405)**	0.043 (0.484)
Child-days/month children helped ADL	6.188 (2.558)**	9.082	6.697 (3.046)**	4.183 (5.206)	11.480 (3.781)***	−5.475 (4.085)
Had ADL help from relatives	−0.031 (0.016)**	0.042	−0.027 (0.018)	−0.057 (0.033)*	−0.037 (0.024)	−0.032 (0.026)
N. relatives helped with ADL	0.178 (0.173)	0.097	0.203 (0.229)	0.178 (0.184)	0.216 (0.390)	0.173 (0.142)

*Notes:* Each observation is an individual-year pair, and the units of analysis are individuals older than 45 and their partners, when one is present, regardless of age. Table shows the coefficients from a regression of the first difference of the dependent variable—displayed in the first column—on the first difference of the health shock measure. **All** shows results for all sample; **Mean** displays the mean of the lagged value of the dependent variable; **Rural** and **Urban** shows results for subsamples of rural and urban households, respectively; **Female** and **Male** shows results for subsamples of female and male respondents, respectively. Health is created by adding six indicator variables for the presence of difficulties with a basic ADL (getting dressed, bathing or showering, eating, getting in and out of bed, using the toilet, and controlling urination and defecation) and dividing the result by six. Therefore, Health ranges from 0 (if one no difficulties with any of the six basic ADLs) to 1 (if one has difficulty with all six basic ADLs). Dependent variables are displayed in the first column. Household size is the number of household members other than one's spouse. Had children co-residing is an indicator for having at least one child co-residing. Lived near children is an indicator for having at least one child living in same city or county as individual/spouse. Had contact with children is an indicator for any weekly contact with a non-coresiding child, either in-person, by phone, text message, mail, or e-mail. Had contact with parents is an indicator for any weekly contact with a non-coresiding parent/in-law. Had ADL help is an indicator for whether individual received any care for basic or instrumental ADLs. Had ADL help from spouse indicates receiving adl/iadl care from spouse/ex-spouse. Days/month spouse helped ADL is the number of days in a month when individual received care for adl/iadl from spouse/ex-spouse. Had ADL help from children indicates receiving adl/iadl care from children/grand-children. Child-days/month spouse helped ADL is the total number of days in a month when individual received care for adl/iadl from children/grand-children. Had ADL help from relatives indicates receiving adl/iadl care from relatives (this includes mother, father, mother-in-law, father-in-law, siblings, siblings of spouse, brother-in-laws, sister-in-laws, or other relatives). N. relatives helped with ADL is the total number of relatives that helped with adl/iadl. All regressions include rural village (or urban community) fixed effects interacted with survey year fixed effects. Demographic controls: sex, education, birth-year indicators, number of children ever born, and rural/urban status. Standard errors are clustered at the household level. Data source: CHARLS 2011, 2013, 2015, and 2018.

* p<0.1,

** p<0.05,

*** p<0.01.

**Table 6:** Health and family mortality

Dep. var.: Change in	All	Mean	Rural	Urban	Female	Male

Mother dead	0.001 (0.007)	0.765	−0.008 (0.009)	0.023 (0.015)	0.006 (0.010)	−0.007 (0.012)
Father dead	−0.004 (0.006)	0.870	−0.003 (0.006)	−0.008 (0.012)	0.003 (0.008)	−0.020 (0.009)**
Daughter dead	0.014 (0.008)*	0.034	0.016 (0.011)	0.010 (0.012)	0.017 (0.011)	0.013 (0.014)
Son dead	0.007 (0.009)	0.050	0.009 (0.011)	−0.002 (0.013)	0.006 (0.012)	0.009 (0.013)
Sister dead	−0.011 (0.013)	0.191	−0.004 (0.015)	−0.030 (0.024)	−0.028 (0.017)	0.015 (0.019)
Brother dead	−0.011 (0.012)	0.255	−0.007 (0.015)	−0.022 (0.023)	−0.016 (0.016)	−0.006 (0.020)
Spouse dead	0.008 (0.007)	0.096	0.008 (0.008)	0.008 (0.013)	0.010 (0.010)	0.007 (0.009)

*Notes:* Each observation is an individual-year pair, and the units of analysis are individuals older than 45 and their partners, when one is present, regardless of age. Table shows the coefficients from a regression of the first difference of the dependent variable—displayed in the first column—on the first difference of the health shock measure. **All** shows results for all sample; **Mean** displays the mean of the lagged value of the dependent variable; **Rural** and **Urban** shows results for subsamples of rural and urban households, respectively; **Female** and **Male** shows results for subsamples of female and male respondents, respectively. Health is created by adding six indicator variables for the presence of difficulties with a basic ADL (getting dressed, bathing or showering, eating, getting in and out of bed, using the toilet, and controlling urination and defecation) and dividing the result by six. Therefore, Health ranges from 0 (if one no difficulties with any of the six basic ADLs) to 1 (if one has difficulty with all six basic ADLs). Mother dead is an indicator for whether the individual's mother is deceased. Father dead is an indicator for whether the individual's father is deceased. Daughter dead is the number of deceased daughters of individual and spouse at the household level. Son dead is the number of deceased daughters of individual and spouse at the household level. Sister dead is the number of deceased sisters of the individual. Brother dead is the number of deceased brothers of the individual. Spouse dead is an indicator for whether spouse is deceased. All regressions include rural village (or urban community) fixed effects interacted with survey year fixed effects. Demographic controls: sex, education, birth-year indicators, number of children ever born, and rural/urban status. Standard errors are clustered at the household level. Data source: CHARLS 2011, 2013, 2015, and 2018.

* p<0.1,

** p<0.05,

*** p<0.01.

**Table 7:** The effects of health on labor supply and earned income

Dep. var.: Change in	All	Mean	Rural	Urban	Female	Male

Currently working	−0.113 (0.018)***	0.666	−0.127 (0.023)***	−0.083 (0.029)***	−0.079 (0.025)***	−0.152 (0.029)***
Hours worked	−3.993 (0.989)***	28.476	−4.321 (1.254)***	−3.273 (1.509)**	−2.424 (1.277)*	−5.568 (1.671)***
Lost work bc sick	0.342 (0.042)***	0.333	0.353 (0.046)***	0.309 (0.103)***	0.324 (0.059)***	0.418 (0.061)***
Earned income	−0.415 (0.101)***	1.826	−0.475 (0.123)***	−0.292 (0.176)*	−0.094 (0.107)	−0.826 (0.196)***
Spouse currently working	−0.014 (0.020)	0.703	−0.018 (0.024)	−0.011 (0.038)	−0.049 (0.027)*	0.014 (0.033)
Spouse hours worked	1.567 (1.264)	30.717	2.104 (1.514)	0.123 (2.338)	0.734 (1.787)	2.325 (1.899)
Spouse income	−0.122 (0.145)	1.934	−0.160 (0.171)	−0.042 (0.275)	−0.357 (0.240)	0.040 (0.171)
Other hh member made income	0.040 (0.019)**	0.296	0.040 (0.023)*	0.036 (0.035)	0.051 (0.026)**	0.018 (0.029)

*Notes:* Each observation is an individual-year pair, and the units of analysis are individuals older than 45 and their partners, when one is present, regardless of age. Table shows the coefficients from a regression of the first difference of the dependent variable—displayed in the first column—on the first difference of the health shock measure. **All** shows results for all sample; **Mean** displays the mean of the lagged value of the dependent variable; **Rural** and **Urban** shows results for subsamples of rural and urban households, respectively; **Female** and **Male** shows results for subsamples of female and male respondents, respectively. Health is created by adding six indicator variables for the presence of difficulties with a basic ADL (getting dressed, bathing or showering, eating, getting in and out of bed, using the toilet, and controlling urination and defecation) and dividing the result by six. Therefore, Health ranges from 0 (if one no difficulties with any of the six basic ADLs) to 1 (if one has difficulty with all six basic ADLs). Dependent variables are displayed in the first column. Currently working indicates whether the respondent engaged in any work in the past year (it takes on value zero if unemployed, retired, or never worked). Hours worked indicates the number of hours per week worked at the main job in the past year (it takes on value zero if currently not working). Lost work bc sick indicates whether a respondent who currently works has missed work because of sickness. Spouse currently working indicates whether the respondent’s spouse engaged in any work in the past year (it takes on value zero if unemployed, retired, or never worked); variable is missing for uncoupled individuals. Earned income is the individual's past year wages and bonus income after tax; we take the log of income and replace it with zero when income is zero or when the individual is currently not working. Spouse hours worked indicates the number of hours per week the individual's spouse worked at the main job in the past year (it takes on value zero if currently not working). Spouse earned income is the individual's spouse's past year wages and bonus income after tax; we take the log of income and replace it with zero when income is zero or when the individual's spouse is currently not working. Other hh member made income is an indicator for whether income from other household members (other than spouse) is positive. All regressions include rural village (or urban community) fixed effects interacted with survey year fixed effects. Demographic controls: sex, education, birth-year indicators, number of children ever born, and rural/urban status. Standard errors are clustered at the household level. Data source: CHARLS 2011, 2013, 2015, and 2018.

* p<0.1,

** p<0.05,

*** p<0.01.

**Table 8:** The effects of health on consumption

Dep. var.: Change in	All	Mean	Rural	Urban	Female	Male

HH per capita income	0.061 (0.132)	7.277	−0.030 (0.154)	0.271 (0.255)	0.105 (0.180)	−0.037 (0.207)
Pension income	−0.074 (0.133)	2.493	−0.026 (0.150)	−0.157 (0.269)	−0.191 (0.171)	0.041 (0.204)
HH per capita food consumption	0.115 (0.101)	7.864	0.154 (0.130)	0.018 (0.142)	0.201 (0.142)	0.064 (0.163)
HH pc monthly non-food cons.	0.087 (0.062)	6.665	0.091 (0.072)	0.090 (0.118)	0.139 (0.076)*	0.083 (0.114)
HH pc annual non-food cons.	0.291 (0.103)***	6.992	0.319 (0.121)***	0.209 (0.197)	0.103 (0.135)	0.566 (0.169)***

*Notes:* Each observation is an individual-year pair, and the units of analysis are individuals older than 45 and their partners, when one is present, regardless of age. Table shows the coefficients from a regression of the first difference of the dependent variable—displayed in the first column—on the first difference of the health shock measure. **All** shows results for all sample; **Mean** displays the mean of the lagged value of the dependent variable; **Rural** and **Urban** shows results for subsamples of rural and urban households, respectively; **Female** and **Male** shows results for subsamples of female and male respondents, respectively. Health is created by adding six indicator variables for the presence of difficulties with a basic ADL (getting dressed, bathing or showering, eating, getting in and out of bed, using the toilet, and controlling urination and defecation) and dividing the result by six. Therefore, Health ranges from 0 (if one no difficulties with any of the six basic ADLs) to 1 (if one has difficulty with all six basic ADLs). Dependent variables are displayed in the first column. HH per capita income is the sum of earnings income (wages and self-employment), pension income, income from government transfers, other income, and total income from other household members, all divided by the number of household members (including respondent and spouse); we take the log of income and replace it with zero when income is zero or negative. Pension income is the individual's income from private and public pensions such as new rural resident pensions, pension subsidy to the oldest old, urban resident’s pension, commercial pension insurance, social insurance, among others; we take the log of income and replace it with zero when income is zero. Total HH per capita consumption is the sum of all consumption activities, divided by the number of household members (including respondent and spouse): food consumption in the past week, non-food consumption in the past month, and other non-food consumption in the past year, and it is calculated as annual expenditures; we take the log of consumption. HH per capita food consumption is the total (per capita) household food expenditure on purchased food and food eaten from own production, meals eaten out, and alcohol/tobacco; it is measured in logs. HH pc monthly non-food cons. is the (per capita) total household non-food expenditure in the last month on utilities, fuels, entertainment, transportation, and daily items, measured in logs. HH pc annual non-food cons. is the total (per capita) household non-food expenditure in the last year on clothing, durable goods, education, donations, long-distance travel, automobiles, maintance and repairs, and medical expenses. All regressions include rural village (or urban community) fixed effects interacted with survey year fixed effects. Demographic controls: sex, education, birth-year indicators, number of children ever born, and rural/urban status. Standard errors are clustered at the household level. Data source: CHARLS 2011, 2013, 2015, and 2018.

* p<0.1,

** p<0.05,

*** p<0.01.

## Footnotes

1 China became an ageing society in 2000, with the proportion of people aged 65 or older nearly doubling in the last 20 years - growing from 7.4% in 2000 to 13.5% in 2020 (Ai et al. (2022), Seventh National Population Census) and to 14.2% in 2021 (Chen et al., 2022).

2 While both the China Health and Nutrition Survey (CHNS) and the China Health and Retirement Longitudinal Study (CHARLS) find declines in ADLs and IADLs over the last 20–30 years (1997 to 2006 in the CHNS and 2011 to 2020 in the CHARLS), there is still a higher prevalence of disability among rural compared to urban older people (Ai et al., 2022).

3 The New Rural Cooperative Medical Scheme was introduced in 2003 in order to provide health insurance to all rural residents and the Urban Resident Basic Medical Insurance Scheme was introduced in 2009 to fill the remaining gap, providing care to urban residents who were not already covered by the Urban Workers Basic Medical Insurance Scheme, which was directly tied to formal employment and only covered the employed individual (Strauss et al., 2010). Coverage has increased from 50% of city dwellers in 2005 to 86% of rural counties (Strauss et al., 2010), and now 96% of Chinese citizens are enrolled in health insurance with the introduction of the Urban Workers Basic Medical Insurance Scheme (Chen et al., 2022). However, there is still segmentation by program type, locality, and occupation; and there are low reimbursement rates for expenses incurred outside of the home (Chen et al., 2022).

4 Previous studies have argued that difficulties with ADLs are good indicators of physical functioning ability in both developed and developing country contexts (Stewart et al., 1990; Strauss et al., 1993; Gertler and Gruber, 2002), and they are particularly reliable measures of elderly health status (Strauss et al., 2010; Genoni, 2012).

5 Instrumental ADLs refer to the following activities: managing money, taking medication, shopping for groceries, preparing meals, cleaning house, and making phone calls.

6 Schaller and Eck (2023) also examines old-age individuals. However, they study the U.S. where downstream intergenerational transfers are the main focus.

7 Altruism as a motive for transfers has been widely studied as a way to understand private inter-vivo transfers in general, and inter-generational support in particular. See Wu (2003) for empirical evidence of altruism in transfers from children to parents. For applications to the Chinese context, see Cai et al. (2006), Oliveira (2016) and Ho (2019). For discussions of motives for transfers beyond altruism see Cox (1987), Cox and Rank (1992), Cox et al. (1998), Laferrère and Wolff (2006).

8 This is known as state dependence, and has been study in the health literature. See Finkelstein et al. (2013), Dobkin et al. (2018) and Blundell et al. (2024) for a formal modeling and empirical tests.

9 CHARLS is supported by Peking University, the National Natural Science Foundation of China, the National Institute on Aging, and the World Bank.

10 More specifically we use the dataset and codebook from Version D as of June 2021. The development of the Harmonized CHARLS was funded by the National Institute on Aging (R01 AG030153, RC2 AG036619, R03 AG043052). For more information, please refer to https://g2aging.org/

11 Because we estimate the model in the first differences, our year units refer to the difference between the current survey year and the previous survey year. Therefore, we have a total of three possible year differences: 2018–2015, 2015–2013, and 2013–2011.

12 This measure has the advantage of being more objective than self-reported measures of (good, fair, or bad) health status and also less susceptible to capturing predicted and endogenous changes in health driven by behavior, such as being diagnosed with major illnesses.

13 The indicator is assigned value one if the individual: has difficulty, but can do it; has difficulty and need help; or cannot do it.

14 If an individual or their spouse have no children or no non-resident child/grandchild, the amount of transfers from children is zero.

15 The summary reported is based on polled data across respondents and survey years. Because we take the first difference, the values are missing for the first survey year, 2011.

16 When calculating the percentage of transfers from parents/in-laws, we consider individuals with at least one parent or parent in law alive.

17 We use the average exchange rate as of 2015.

18 We consider six basic ADLs: getting dresses, bathing or showering, eating, getting in and out of bed, using the toilet, and controlling urination and defecation.

19 This finding is in contrast with Giles and Mu (2007).

20 IADL refers to instrumental activities of daily living: managing money, taking medications, shopping for groceries, preparing meals, cleaning house and making phone calls.

21 For studies on the importance of spouses as main source of instrumental care, and the impact of caring for an ill spouse on an individual’s own health, see Chen et al. (2018), Chen et al. (2022), and Ai et al. (2022).
